# The Effects of Omega-3 Fatty Acid on Vitamin D Activation in Hemodialysis Patients: A Pilot Study

**DOI:** 10.3390/md13020741

**Published:** 2015-01-28

**Authors:** Su Mi Lee, Young Ki Son, Seong Eun Kim, Won Suk An

**Affiliations:** 1 Department of Internal Medicine, Dong-A University, Busan 602-715, Korea; E-Mails: promise131@hanmail.net (S.M.L.); kidney@dau.ac.kr (Y.K.S.); sekim@dau.ac.kr (S.E.K.); 2 Institute of Medical Science, Dong-A University College of Medicine, Busan 602-715, Korea

**Keywords:** omega-3 fatty acid, 25-hydroxyvitamin D, 1,25-dihydroxyvitamin D, vitamin D, hemodialysis

## Abstract

The high incidence of cardiovascular disease and vitamin D deficiency in chronic kidney disease patients is well known. Vitamin D activation by omega-3 fatty acid (FA) supplementation may explain the cardioprotective effects exerted by omega-3 FA. We hypothesized that omega-3 FA and 25-hydroxyvitamin D (25(OH)D) supplementation may increase 1,25-dihydroxyvitamin D (1,25(OH)_2_D) levels compared to 25(OH)D supplementation alone in hemodialysis (HD) patients that have insufficient or deficient 25(OH)D levels. We enrolled patients that were treated for at least six months with 25(OH)D < 30 ng/mL (NCT01596842). Patients were randomized to treatment for 12 weeks with cholecalciferol supplemented with omega-3 FA or a placebo. Levels of 25(OH)D and 1,25(OH)_2_D were measured after 12 weeks. The erythrocyte membrane FA contents were also measured. Levels of 25(OH)D were increased in both groups at 12 weeks compared to baseline. The 1,25(OH)_2_D levels at 12 weeks compared to baseline showed a tendency to increase in the omega-3 FA group. The oleic acid and monounsaturated FA content decreased, while the omega-3 index increased in the omega-3 FA group. Omega-3 FA supplementation may be partly associated with vitamin D activation, although increased 25(OH)D levels caused by short-term cholecalciferol supplementation were not associated with vitamin D activation in HD patients.

## 1. Introduction

Chronic kidney disease (CKD) is recognized as a public health problem, and the incidence rate is increasing each year. Decreased renal function is a powerful predictor of cardiovascular morbidity, mortality and all-cause mortality [1,2]. Cardiovascular disease (CVD) is also a primary cause of mortality in patients that have decreasing renal function [3]. This probably relates to inflammation, malnutrition, atherosclerosis, dyslipidemia and vascular calcification [4,5,6,7]. In addition, vitamin D deficiency (VDD) is a known risk factor for CVD in CKD patients [8]. VDD is associated with CVD even among the general population [9]. Several studies have shown a close association between VDD and increased risk of CVD among CKD patients [10,11]. In particular, VDD was associated with an increased risk of CVD and mortality in hemodialysis (HD) patients [12,13,14].

Vitamin D is hydroxylated to 25-hydroxyvitamin D (25(OH)D) in the liver and is then converted to 1,25-dihydroxyvitamin D (1,25(OH)_2_D) by the enzyme 1α-hydroxylase in the kidney. Because decreased renal function may induce suppression of 1α-hydroxylase activity, active vitamin D levels are lower in patients with CKD than in the general population [15]. Expression of 1α-hydroxylase is predominantly in the proximal renal tubular cells [16]. However, this enzyme is also expressed in extra-renal tissues, such as the skin, lymph nodes, placenta, prostate, breast and colon [17,18]. A previous study has reported that extra-renal sources of 1,25(OH)_2_D can be increased to normal serum 1,25(OH)_2_D levels in HD patients after administration of high doses of 25(OH)D [19]. We recently demonstrated that 1,25(OH)_2_D levels significantly increased in dialysis patients compared to baseline after three months of omega-3 fatty acid (FA) supplementation without 25(OH)D administration [20]. In uremic condition, the levels of enzymes related with lipid metabolism are up- or down-regulated compared to normal condition. Omega-3 FA, having an anti-inflammatory effect and reducing oxidative stress, may modify uremic condition and possibly regulate enzyme levels. The levels of 1α-hydroxylase and 24-hydroxylase affecting 1,25(OH)_2_D levels also change in uremic condition. Therefore, we suggest that 1,25(OH)_2_D levels may be changed by omega-3 FA supplementation in uremic condition.

In this study, we hypothesized that cholecalciferol with omega-3 FA supplementation may increase the levels of 25(OH)D and 1,25(OH)_2_D beyond what is achieved with 25(OH)D supplementation alone in HD patients with insufficient or deficient 25(OH)D levels. In addition, we evaluated the effects of omega-3 FA on the change of erythrocyte membrane FA contents in HD patients.

## 2. Results

### 2.1. Baseline Characteristics

The baseline characteristics of the study participants are listed in Table 1. Two patients withdrew from the study. They were admitted to the hospital because of vaginal bleeding or general weakness. Therefore, there were 15 HD patients (eight in the cholecalciferol with omega-3 FA group and seven in the cholecalciferol with olive oil group) that completed the study. The mean age of the patients was 62.1 ± 7.9 years, and 33.3% of the study population consisted of men. There were no significant differences between the cholecalciferol with olive oil and the cholecalciferol with omega-3 FA groups.

**Table 1:** Clinical blood biochemical analyses of the subjects.

	Cholecalciferol with Olive Oil (*n* = 7)	Cholecalciferol with Omega-3 FA (*n* = 8)	*p*-Value ^1^
Age (years)	64.4 ± 8.5	60.0 ± 7.3	0.397
Male, *n* (%)	3 (42.9%)	2 (25%)	0.464
DM, *n* (%)	4 (57.1%)	7 (87.5%)	0.185
25(OH)D (ng/mL)	10.2 ± 1.9	10.2 ± 4.0	0.613
1,25(OH)_2_D (pg/mL)	24.1 ± 11.1	17.7 ± 8.2	0.336
Total Cholesterol (mg/dL)	155.3 ± 27.9	153.3 ± 21.0	0.694
Triglyceride (mg/dL)	158.4 ± 89.7	174.8 ± 90.3	0.732
HDL (mg/dL)	40.7 ± 4.7	48.9 ± 9.0	0.189
LDL (mg/dL)	90.6 ± 21.5	81.4 ± 19.0	0.463
Glucose (mg/dL)	86.7 ± 20.9	130.9 ± 58.1	0.152
Calcium (mg/dL)	8.7 ± 0.7	9.5 ± 1.1	0.189
Phosphorus (mg/dL)	5.0 ± 1.7	4.6 ± 1.7	0.694
PTH (pg/mL)	560.8 ± 236.5	325.9 ± 338.7	0.094
BUN (mg/dL)	66.3 ± 17.6	56.8 ± 15.8	0.336
Creatinine (mg/dL)	10.3 ± 3.0	9.6 ± 2.6	0.336
Hemoglobin (g/dL)	10.1 ± 1.0	10.9 ± 1.3	0.281
Albumin (mg/dL)	4.0 ± 0.2	3.9 ± 0.2	0.463
CRP (mg/dL)	1.2 ± 1.3	0.2 ± 0.1	0.014
Iron (μg/dL)	61.7 ± 12.3	81.3 ± 30.3	0.298
TIBC (μg/dL)	222.7 ± 26.7	241.4 ± 26.2	0.203
Ferritin (μg/dL)	305.0 ± 289.3	215.1 ± 91.6	0.908
Calcium load (g/day)	2354.9 ± 2023.1	2342.8 ± 1018.0	0.867
Phosphate binder (%)	5 (71.4%)	8 (100.0%)	0.104
Vitamin D medication (%)	4 (57.1%)	2 (25.0%)	0.205
Cinacalcet medication (%)	1 (14.3%)	1 (12.5%)	0.919

Data are expressed as the means ± SD. ^1^ *p*-value for the nonparametric Mann–Whitney *U*-test comparing baseline data between the cholecalciferol with olive oil group and cholecalciferol with omega-3 FA group. The difference in frequency was tested using Pearson *X*^2^. Abbreviations: DM, diabetes mellitus; HDL, high-density lipoprotein cholesterol; LDL, low-density lipoprotein cholesterol; PTH, parathyroid hormone; BUN, blood urea nitrogen; CRP, C-reactive protein; TIBC, total iron binding capacity.

### 2.2. Diet Consumption Data

The results for the survey of food consumption are displayed in Table 2. There were no significant differences in the diet consumption data between the cholecalciferol with olive oil group and the cholecalciferol with omega-3 FA group. There were also no significant differences compared to baseline in the diet consumption data between the cholecalciferol with olive oil group and the cholecalciferol with omega-3 FA group after 12 weeks.

**Table 2:** Dietary consumption of foods and nutrients.

	Cholecalciferol with Olive Oil		Cholecalciferol with Omega-3 FA	
	Baseline	12 Weeks	Baseline	12 Weeks
Kcal (kcal)	1588.9 ± 435.9	1371.8 ± 685.2	1316.2 ± 383.1	1105.7 ± 521.6
Animal protein (g)	19.7 ± 11.9	21.3 ± 18.7	16.3 ± 6.8	17.6 ± 13.4
Vegetable protein (g)	26.9 ± 8.0	24.8 ± 12.5	24.4 ± 7.6	20.9 ± 11.4
Animal lipid (g)	12.9 ± 7.7	14.3 ± 11.8	9.5 ± 4.1	10.8 ± 8.1
Vegetable lipid (g)	10.6 ± 7.0	12.0 ± 8.9	8.6 ± 3.4	10.6 ± 4.6
Carbohydrate (g)	292.3 ± 64.6	236.3 ± 107.2	245.8 ± 64.6	189.8 ± 80.4
Fiber (g)	12.1 ± 4.9	13.0 ± 7.6	13.4 ± 4.2	12.1 ± 6.1
Retinol (μg)	108.6 ± 97.4	135.1 ± 77.6	67.5 ± 45.8	87.9 ± 40.6
Niacin (mg)	9.5 ± 3.6	9.2 ± 5.2	9.1 ± 3.1	7.6 ± 4.8
Vitamin E (mg)	9.5 ± 4.8	8.3 ± 4.9	8.4 ± 2.0	8.0 ± 3.9
Cholesterol (mg)	213.8 ± 154.3	237.7 ± 186.6	148.7 ± 73.5	236.0 ± 128.9

Data are expressed as the means ± SD. The nonparametric Wilcoxon exact rank sum test was used to compare baseline data with 12-week data.

### 2.3. Changes in Biochemical Data

**Figure 1:**
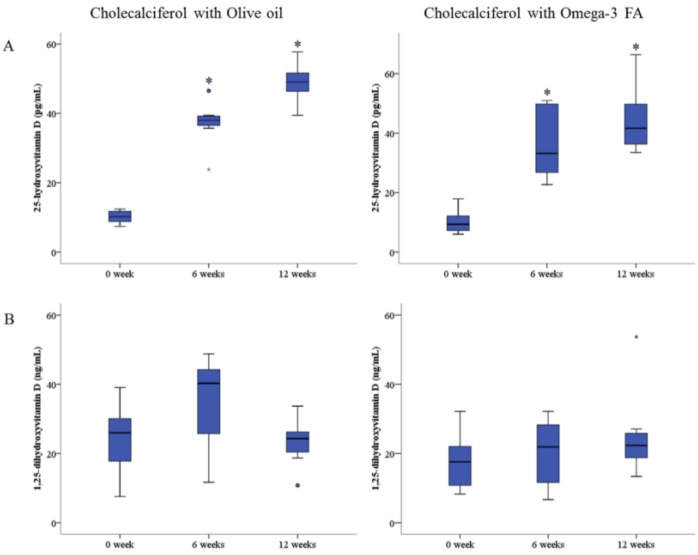
(**A**) Change of 25-hydroxyvitamin D level by cholecalciferol with omega-3 FA supplementation. (**B**) Change of 1,25-dihydroxyvitamin D level by cholecalciferol with omega-3 FA supplementation. * *p*-value < 0.05 (mean values are significantly different from baseline). Repeated-measure analysis of variance was used to compare baseline data with six-week and 12-week data.

**Figure 2:**
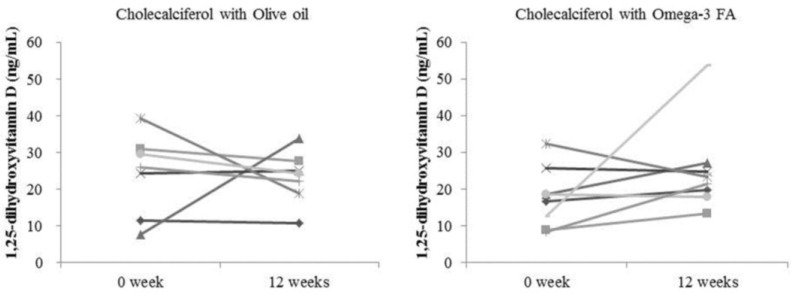
Change in 1,25-dihydroxyvitamin D level according to each patient.

Changes in the biochemical data after 12 weeks compared to baseline are shown in Table 3. The levels of 25(OH)D significantly increased in both the cholecalciferol with olive oil group and the cholecalciferol with omega-3 FA group after 12 weeks compared to baseline (48.9 ± 5.8 pg/mL and 10.2 ± 1.9 pg/mL, *p* = 0.018 *vs.* 44.4 ± 10.8 pg/mL and 10.2 ± 4.0 pg/mL, *p* = 0.012, respectively; Figure 1A). However, the level of 1,25(OH)_2_D was not significantly increased in either the cholecalciferol with olive oil group or the cholecalciferol with omega-3 FA group after 12 weeks compared to baseline (23.2 ± 7.2 ng/mL and 24.1 ± 11.1 ng/mL, *p* = 0.398 *vs.* 25.1 ± 12.3 ng/mL and 17.7 ± 8.2 ng/mL, *p* = 0.208, respectively; Figure 1B). Although the change in the level of 1,25(OH)_2_D was not statistically significant, the level showed a tendency to increase in the group that received cholecalciferol supplemented with omega-3 FA (Figure 2). Calcium, phosphorous and parathyroid hormone (PTH) levels were not significantly altered, but the levels of high-density lipoprotein cholesterol (HDL) and low-density lipoprotein cholesterol (LDL) were significantly lower in the cholecalciferol with omega-3 FA group after 12 weeks compared to baseline (*p* = 0.024 and *p* = 0.025, respectively). Docosahexaenoic acid (DHA), among omega-3 FA, was not related with the ratio of 1,25(OH)_2_D to the 25(OH)D and 1,25(OH)_2_D levels at baseline, but DHA was significantly correlated with the ratio of 1,25(OH)_2_D to 25(OH)D (Spearman’s correlation coefficient (*r* = 0.543, *p* = 0.037) and partly correlated with 1,25(OH)_2_D (*r* = 0.507, *p* = 0.054) in the 15 patients’ correlation analysis after 12 weeks.

**Table 3:** Changes in biochemical data.

	Cholecalciferol with Olive Oil		Cholecalciferol with Omega-3 FA	
	Baseline	12 Weeks	Baseline	12 Weeks
25(OH)D (ng/mL)	10.2 ± 1.9	48.9 ± 5.8 *	10.2 ± 4.0	44.4 ± 10.8 *
1,25(OH)_2_D (pg/mL)	24.1 ± 11.1	23.2 ± 7.2	17.7 ± 8.2	25.1 ± 12.3
1,25(OH)_2_D/25(OH)D	2.4 ± 1.1	0.5 ± 0.2 ^*^	1.8 ± 0.5	0.6 ± 0.3 *
Total Cholesterol (mg/dL)	155.3 ± 27.9	159.7 ± 29.8	153.3 ± 21.0	139.0 ± 39.0
Triglyceride (mg/dL)	158.4 ± 89.7	123.3 ± 56.9	174.8 ± 90.3	156.0 ± 120.7
HDL (mg/dL)	40.7 ± 4.7	43.1 ± 5.5	48.9 ± 9.0	41.5 ± 6.5 *
LDL (mg/dL)	90.6 ± 21.5	90.7 ± 26.0	81.4 ± 19.0	68.8 ± 28.6 *
Glucose (mg/dL)	84.7 ± 20.9	84.1 ± 21.2	130.9 ± 58.1	128.4 ± 103.9
BUN (mg/dL)	66.3 ± 17.6	71.0 ± 19.7	56.8 ± 15.8	65.9 ± 14.1
Creatinine (mg/dL)	10.3 ± 3.0	10.9 ± 3.1 *	9.6 ± 2.6	10.6 ± 1.7
Hemoglobin (g/dL)	10.1 ± 1.0	10.3 ± 0.7	10.9 ± 1.3	11.0 ± 1.1
Albumin (mg/dL)	4.0 ± 0.2	4.2 ± 0.2	3.9 ± 0.2	3.9 ± 0.2
CRP (mg/dL)	1.2 ± 1.3	0.9 ± 1.0 *	0.2 ± 0.1	0.7 ± 1.5
Fetuin-A (μg/dL)	185.7 ± 31.9	180.3 ± 31.9	220.8 ± 49.9	223.4 ± 67.8
FGF-23 (pg/mL)	1737.2 ± 2344.8	2001.8 ± 2176.1	1879.5 ± 1563.2	2534.5 ± 2270.5
Calcium (mg/dL)	8.7 ± 0.7	9.3 ± 0.8	9.5 ± 1.1	9.3 ± 1.2
Phosphorus (mg/dL)	5.0 ± 1.7	5.4 ± 1.9	4.6 ± 1.7	5.1 ± 1.4
PTH (pg/mL)	560.8 ± 236.5	381.5 ± 215.6	325.9 ± 338.7	289.2 ± 273.0
Iron (μg/dL)	61.7 ± 12.3	67.1 ± 28.1	81.3 ± 30.3	54.6 ± 19.9 *
TIBC (μg/dL)	222.7 ± 26.7	235.3 ± 24.1	241.4 ± 26.2	219.3 ± 23.6 *
Ferritin (μg/dL)	305.0 ± 289.3	305.2 ± 232.8	215.1 ± 91.6	319.7 ± 174.4

Data are expressed as the means ± SD. The nonparametric Wilcoxon exact rank sum test was used to compare baseline data with 12-week data. * *p*-value < 0.05 (mean values are significantly different from baseline). Abbreviations: HDL, high-density lipoprotein cholesterol; LDL, low-density lipoprotein cholesterol; BUN, blood urea nitrogen; CRP, C-reactive protein; FGF, fibroblast growth factor; PTH, parathyroid hormone; TIBC, total iron binding capacity.

### 2.4. Changes in Erythrocyte Membrane FA Content

The erythrocyte membrane contents of eicosapentaenoic acid (EPA) and DHA and the omega-3 index were significantly increased in the cholecalciferol with omega-3 FA group after 12 weeks compared to baseline (*p* = 0.012, *p* = 0.012 and *p* = 0.012, respectively; Table 4). The monounsaturated FA and oleic acid contents of the erythrocyte membrane were significantly lower in the cholecalciferol with omega-3 FA group after 12 weeks compared to baseline (*p* = 0.012 and *p* = 0.017, respectively). The erythrocyte membrane arachidonic acid (AA) content was not significantly altered, but the ratio of AA to EPA was significantly lower in the cholecalciferol with omega-3 FA group after 12 weeks compared to baseline (*p* = 0.779 and *p* = 0.012, respectively).

**Table 4:** Changes in erythrocyte membrane fatty acids content.

	Cholecalciferol with Olive Oil		Cholecalciferol with Omega-3 FA	
	Baseline	12 Weeks	Baseline	12 Weeks
Saturated	47.0 ± 8.6	40.4 ± 0.5	45.1 ± 9.8	41.4 ± 1.2
Myristic	0.5 ± 0.2	0.3 ± 0.1 *	0.5 ± 0.1	0.4 ± 0.1
Palmitic	25.9 ± 4.4	21.9 ± 0.5 *	25.4 ± 5.1	23.1 ± 1.4
Stearic	20.0 ± 4.2	17.7 ± 0.6	18.8 ± 4.7	17.4 ± 0.6
Lignoceric	0.6 ± 0.2	0.4 ± 0.1 *	0.5 ± 0.2	0.5 ± 0.2
Monounsaturated	17.8 ± 1.9	16.1 ± 0.9 *	17.5 ± 1.5	15.9 ± 1.0 *
Palmitoleic	0.8 ± 0.4	0.6 ± 0.2	0.7 ± 0.2	0.6 ± 0.2
Oleic	16.0 ± 1.6	14.6 ± 0.8	15.8 ± 1.5	14.5 ± 1.0 *
Polyunsaturated	34.0 ± 10.3	42.5 ± 0.7	36.2 ± 11.1	41.7 ± 1.5
Omega-6	23.3 ± 5.0	28.2 ± 2.8	25.8 ± 7.0	24.6 ± 2.3
Linoleic	9.5 ± 1.3	9.2 ± 1.5	10.0 ± 2.1	9.3 ± 1.4
AA	10.0 ± 4.0	14.1 ± 1.2 *	11.2 ± 4.6	11.3 ± 1.8
Omega-3	10.8 ± 5.7	14.3 ± 2.3	10.4 ± 4.5	17.1 ± 2.7 *
Alpha-linolenic	0.4 ± 0.2	0.2 ± 0.1 *	0.2 ± 0.1	0.2 ± 0.1
EPA	1.6 ± 0.9	2.0 ± 0.9	1.3 ± 0.5	3.9 ± 1.4 *
DHA	6.9 ± 3.9	9.2 ± 1.5	6.6 ± 2.9	9.3 ± 1.2 *
Omega-3 index	8.4 ± 4.7	11.2 ± 2.1	7.8 ± 3.4	13.2 ± 2.2 *
AA/EPA	8.2 ± 4.6	8.9 ± 6.3	9.2 ± 3.6	3.5 ± 2.3 *
Omega-6/Omega-3	2.7 ± 1.3	2.1 ± 0.6	2.8 ± 1.1	1.5 ± 0.4 *
Total *trans*-fatty acid	0.93 ± 0.24	0.91 ± 0.12	0.91 ± 0.24	0.86 ± 0.14
*Trans*-palmitoleic acid	0.18 ± 0.06	0.16 ± 0.06	0.22 ± 0.03	0.16 ± 0.05 *
*Trans*-oleic acid	0.62 ± 0.18	0.53 ± 0.14	0.57 ± 0.17	0.54 ± 0.12
*Trans*-linoleic acid	0.31 ± 0.11	0.38 ± 0.17	0.34 ± 0.10	0.32 ± 0.08
18:2 *n*6tt	0.06 ± 0.03	0.10 ± 0.09	0.07 ± 0.06	0.05 ± 0.03
18:2 *n*6ct	0.10 ± 0.04	0.11 ± 0.02	0.05 ± 0.03	0.13 ± 0.06
18:2 *n*6tc	0.15 ± 0.07	0.16 ± 0.08	0.17 ± 0.03	0.14 ± 0.04

Data are expressed as the means ± SD. The nonparametric Wilcoxon exact rank sum test was used to compare baseline data with 12-week data. * *p*-value <0.05 (mean values are significantly different from baseline). Abbreviations: AA, arachidonic acid; EPA, eicosapentaenoic acid; DHA, docosahexaenoic acid.

## 3. Discussion

In this study, we found that the levels of 25(OH)D were significantly increased by cholecalciferol supplemented with omega-3 FA or olive oil, but that the levels of 1,25(OH)_2_D were not significantly altered in either group. The 1,25(OH)_2_D levels showed a tendency to increase in the cholecalciferol with omega-3 FA group, but they did not change in the cholecalciferol with olive oil group. While one patient showed a decrease and two patients showed no change in the cholecalciferol with omega-3 FA group, three patients showed a decrease and three patients showed no change in the cholecalciferol with olive oil group. The ratio of 1,25(OH)_2_D to 25(OH)D, which reflects the activation of 1α-hydroxylase, was higher in the cholecalciferol with omega-3 FA group compared to the olive oil group, but was not significant. However, DHA was significantly correlated with the ratio of 1,25(OH)_2_D to 25(OH)D after the 12-week intervention with omega-3 FA and olive oil. This may suggest that omega-3 FA supplementation, including DHA, may be better than the control treatment in terms of regulating the levels of 1,25(OH)_2_D. However, it is not clear that omega-3 FA may have a protective effect against CVD through partial activation of 1α-hydroxylase. This is the first study to evaluate the potential of cholecalciferol with omega-3 FA supplementation in HD patients with insufficient or deficient 25(OH)D levels. Additional prospective studies that are longer and larger than the current study are needed to confirm our findings.

Although a growing number of studies have reported that VDD is a risk factor for CVD, the mechanisms by which vitamin D functions to prevent or treat CVD are unclear. Results from both animal and clinical studies have provided evidence to support a potential cardioprotective effect of vitamin D by inhibition of renin expression, inflammation, vascular smooth muscle cell proliferation and adhesion protein expression in endothelial cells through vitamin D receptor activation [21,22,23,24]. Activated 1,25(OH)_2_D levels are primarily regulated by renal production in healthy individuals and by extra-renal production in end-stage renal disease patients. Previously, extrarenal production of 1,25(OH)_2_D has been observed in anephric HD patients [25]. Several studies have reported that cholecalciferol can increase 25(OH)D and 1,25(OH)_2_D levels in HD patients [19,26]. However, in this study, omega-3 FA supplementation did not result in a substantial increase in the levels of 1,25(OH)_2_D.

There are several possible explanations for the weak effects of omega-3 FA. One possibility is that the treatment periods were insufficient and the sample size was too small. Production of 1,25(OH)_2_D depends on the presence of sufficient levels of the substrate 25(OH)D [27]. In addition, serum 25(OH)D levels decrease over time [28]. Therefore, although an increase in serum 25(OH)D levels was observed in both groups after three months, the levels of 25(OH)D and the treatment periods may not have been adequate to increase the levels of 1,25(OH)_2_D.

Another possibility is that medications could affect vitamin D activation. In this study, we did not exclude patients that were taking cinacalcet, active vitamin D and phosphate binders. Because the doses of active vitamin D and phosphate binders were not modified during the study, the presence of these drugs would not affect the results. However, cinacalcet reduces PTH and calcium levels, thereby regulating the calcium-sensing receptor in the parathyroid gland, and this may subsequently reduce 1,25(OH)_2_D levels. In this study, two patients were taking cinacalcet. In one patient, the levels of 1,25(OH)_2_D increased, and in the other patient, the levels decreased. The relationship between these medications and activation of vitamin D should be evaluated.

The association between the erythrocyte membrane contents of omega-3 FA and the risk of CVD is commonly recognized [29]. In this study, the FA contents in the erythrocyte membrane, including oleic acid, EPA and DHA, were altered by omega-3 FA supplementation. We previously found that the levels of EPA and DHA in erythrocytes were increased, while the levels of oleic acid were decreased by omega-3 FA supplementation in dialysis patients [20,30]. Decreasing oleic acid content is a promising strategy for preventing CVD, because higher erythrocyte membrane oleic acid content is related to acute coronary syndrome and diabetes [31,32,33,34]. Interestingly, the erythrocyte membrane contents of EPA, DHA and oleic acid tended to change in the olive oil group. This suggests that a high dose of olive oil also could change the erythrocyte membrane FA contents toward cardio-protection. These modifications of the erythrocyte membrane contents may be more important findings for cardioprotection than vitamin D activation, because changes of erythrocyte membrane contents are too prominent.

Consumption of *trans*-fatty acids (TFA) may have deleterious effects on cardiac health. Higher intake of TFA is linked to a higher risk of CVD [35,36]. Several previous studies have analyzed the consumption of total TFA, but more recent studies have paid particular attention to several types of TFA, such as *trans*-isomers of oleic acid (*trans*-18:1), *trans*-isomers of linoleic acid (*trans*-18:2) and *trans*-isomers of palmitoleic acid (*trans*-16:1). However, whether different types of TFA have different effects on the heart is unclear, recent data indicates that higher levels of *trans*-linoleic acid and lower levels of *trans*-oleic acid are associated with a higher risk of fatal ischemic heart disease and sudden cardiac death [37,38]. *Trans*-palmitoleic acid is associated with metabolic events, including higher LDL levels and lower triglyceride levels, insulin resistance and incident diabetes [39,40]. However, few studies have analyzed the association between *trans*-palmitoleic acid and the risk of CVD. In this study, erythrocyte membrane FA contents, including *trans*-oleic and *trans*-linoleic, were not altered, but the *trans*-palmitoleic acid content decreased significantly after omega-3 FA supplementation. These results support the need for additional studies to elucidate the potential effects of omega-3 FA on cardiac health according to TFA subtype.

Vascular calcification and arterial stiffening are independent predictors of cardiovascular mortality in CKD patients. Fetuin-A is a 59-kDa glycoprotein that is secreted by the liver [41,42]. It is an important inhibitor of vascular calcification [43]. In the general population, high fetuin-A levels may link fat accumulation in the liver, insulin resistance, metabolic syndrome and an increased risk of myocardial infarction and ischemic stroke [44,45,46]. On the other hand, low fetuin-A levels are associated with malnutrition, inflammation and atherosclerosis, as well as with increased cardiovascular and all-cause mortality in dialysis patients [47,48]. We previously demonstrated that fetuin-A levels were increased after omega-3 FA supplementation in dialysis patients [20]. Unfortunately, we found that fetuin-A levels were not altered by cholecalciferol supplemented with omega-3 FA. This contradictory result may be explained by malnutrition. In the current study, nutritional status was inferior overall, especially in the omega-3 FA group, compared to our previous study, despite the fact that there were no significant changes in both groups [20]. We suspect that decreased levels of total cholesterol, HDL, LDL, iron and total iron binding capacity (TIBC) may be related with nutrition after cholecalciferol supplemented with omega-3 FA. Further studies are needed to demonstrate the effects of cholecalciferol supplemented with omega-3 FA on fetuin-A levels and nutrition in patients with renal dysfunction.

Our study has several limitations. First, the number of patients enrolled in the study was small, and the study took place over a shorter time period compared to other studies. Therefore, the power of the study was limited. Second, we could not control for medications that can affect vitamin D activation. Despite these limitations, we found that cholecalciferol clearly increased 25(OH)D levels without increasing calcium and phosphorus levels in HD patients with insufficient or deficient 25(OH)D levels. Omega-3 FA supplementation may be partly related to vitamin D activation, although increased 25(OH)D levels caused by short-term cholecalciferol supplementation were not related to vitamin D activation in HD patients. In addition, cholecalciferol with omega-3 FA or olive oil can modify the erythrocyte membrane FA content, including oleic acid reduction related with cardioprotection. Further large, prospective studies are necessary to elucidate the effects of omega-3 FA on vitamin D activation and prevention of CVD.

## 4. Materials and Methods

### 4.1. Study Design and Patients

We performed a randomized, double-blind, placebo-controlled study in a single Dong-A University dialysis center (Busan, Korea) between May 2012, and December 2012 (NCT01596842). Seventeen patients who were treated with HD for at least 6 months with 25(OH)D levels <30 pg/mL were included. The exclusion criteria were the following: patients with a history of active infection within 3 months, fish oil or omega-FA supplementation within 3 months, a history of fish, gelatin and/or omega-3 FA allergies, a history of hospital admission within 3 months, a history of bleeding within 3 months, thrombocytopenia, current use of warfarin, an albumin level <3.0 g/dL and malignancy and/or liver cirrhosis. After exclusion, 15 patients were enrolled in this study.

The patients included in the study were randomized to treatment for 12 weeks with omega-3 FA (Omacor, 2.4 g/day; Pronova Biocare, Sandefjord, Norway) or a placebo (olive oil; 2.4 g/day; Suheung Company, Seoul, Korea). There were 460 mg of EPA and 380 mg of DHA in 1 g of Omacor. The omega-3 FA dose was based on a prior study in which at least 2.4 g/d of omega-3 FA supplementation was used in dialysis patients. The nurse administered cholecalciferol (Solgar, Leonia, NJ, USA) during the first dialysis session of the week for a total of 12 weeks. The dose of cholecalciferol was decided as follows: if the baseline 25(OH)D levels were <15 ng/mL, 50,000 IU/week were provided, and if the baseline 25(OH)D levels were <30 ng/mL, 10,000 IU/week were provided. A random number table was used for the randomization. The enrolled patients received regular HD three times a week using a bicarbonate-based dialysate and polysulfone dialyzers (Fresenius, Bad Homburg, Germany).

Informed consent was obtained from all enrolled patients. The study was approved by the Dong-A University Hospital Institutional Review Board. This study was conducted according to the Helsinki Declaration.

### 4.2. Survey of Food Consumption

A survey of food consumption was conducted on the enrolled patients to evaluate the average frequency and portion size of food consumption. The survey was performed at the time the study was initiated and again after 12 weeks. A semi-quantitative, food frequency questionnaire, including 121 foods, was used, as was used in the Korean Cancer Research Survey [49]. To estimate the portion size, three-dimensional food models and full-scale photographs were used. Nutrient intake was estimated using the Computer-Aided Nutritional Analysis Program (Can-Pro 3.0, The Korean Nutrition Society, Seoul, Korea), which provides 1823 food items.

### 4.3. Laboratory Measurements

During the survey, blood samples from each participant were obtained before HD. The blood samples were processed, immediately refrigerated and then stored at −70 °C until analysis. Serum levels of hemoglobin, glucose, blood urea nitrogen, creatinine, albumin, calcium, phosphorus, PTH, C-reactive protein, iron, TIBC, ferritin, total cholesterol, triglyceride, HDL and LDL were analyzed with a routine automated machine at Dong-A University Hospital. Serum 25(OH)D and 1,25(OH)_2_D levels were assessed using a radioimmunoassay kit (DiaSorin Inc. Stillwater, MN, USA). Fetuin-A (Bio Vendor Laboratory Medicine Inc., Brno, Czech Republic) and fibroblast growth factor-23 (Millipore, St Charles, MO, USA) were measured by the enzyme-linked immunosorbent assay.

### 4.4. Gas Chromatography

Erythrocyte membrane FA content was analyzed using methods reported previously [20,30]. Isolated erythrocytes were methylated by the addition of boron trifluoride methanol-benzene for 10 min at 100 °C. Fatty acid methyl esters were analyzed by gas chromatography (Shimadzu 2010AF; Shimadzu Scientific Instrument, Kyoto, Japan) with a 100-m SP2560 capillary column (Supelco, Bellefonte, PA, USA). Fatty acids were identified by comparison with known standards (GLC-727; Nu-Chek Prep, Elysian, MN, USA). The omega-3 index is a measure of EPA and DHA in erythrocyte membranes. Erythrocyte membrane FA content is expressed as a weight percentage.

### 4.5. Statistical Analysis

We calculated that a sample size of 10 patients per group was needed to achieve at least 80% power, to detect an absolute difference in 1,25(OH)_2_D of 12 pg/mL (SD 10 pg/mL) between the omega-3 FA group and the placebo group at a two-sided significance level of 0.05 and assuming a dropout rate of 20% [20].

The data are presented as the mean ± the standard deviation (SD) or frequency. The characteristics were analyzed using the Mann–Whitney *U*-test or Wilcoxon exact rank sum test for non-parametric data and the chi-squared test for the categorical variables. Repeated-measure analysis of variance was used to compare that data acquired at Weeks 0, 6 and 12 of the study. The Spearman analysis was used for the correlations with 1,25(OH)_2_D and the ratio of 1,25(OH)_2_D to 25(OH)D. All of the analyses were performed using the SPSS software (SPSS version 18.0, Chicago, IL, USA). A *p*-value of less than 0.05 was considered statistically significant.

## 5. Conclusions

Omega-3 FA supplementation may be partly related to vitamin D activation, although increased 25(OH)D levels caused by short-term cholecalciferol supplementation were not related to vitamin D activation in HD patients.
